# Autoantibodies as potential biomarkers for breast cancer

**DOI:** 10.1186/bcr2091

**Published:** 2008-05-07

**Authors:** Li Zhong, Kun Ge, Jin-chi Zu, Long-hua Zhao, Wei-ke Shen, Jian-fei Wang, Xiao-gang Zhang, Xu Gao, Wanping Hu, Yun Yen, Kemp H Kernstine

**Affiliations:** 1 Department of Molecular Biology, Hebei University College of Life Sciences, 180 Wusi Road, Baoding 071002, China; 2 Department of Clinical and Molecular Pharmacology, City of Hope and Beckman Research Institute, 1500 Duarte Road, Duarte, CA 91010, USA; 3 Thoracic Surgery & Lung Cancer Program, City of Hope and Beckman Research Institute, 1500 Duarte Road, Duarte, CA 91010, USA; 4 Department of Thoracic Surgery, Hebei University Affiliated Hospital, 320 Yuhua Road, Baoding 071000, China; 5 Teaching and Research Department, China University of Geosciences, Great Wall College, 1698 S. Second Circle Road, Baoding 071001, China; 6 Human Biology Program, Michigan State University College of Natural Science, 103 Natural Science Building, East Lansing, MI 48824, USA; 7 Hematology and Oncology, Kaiser Permanente Health Care, 2295 S. Vineyard, Ontario, CA 91761, USA

## Abstract

**Introduction:**

Only a limited number of tumor markers for breast cancer are currently available. Antibodies to tumor-associated proteins may expand the number of available tumor markers for breast cancer and may be used together in a serum profile to enhance sensitivity and specificity.

**Methods:**

In the present study, we interrogated a breast cancer cDNA T7 phage library for tumor-associated proteins using biopan enrichment techniques with sera from normal individuals and from breast cancer patients. The enrichment of tumor-associated proteins after biopanning was tested using a plaque-lift assay and immunochemical detection. The putative tumor-associated phage clones were collected for PCR and sequencing analysis. Unique and open reading frame phage-expressed proteins were then used to develop phage protein ELISAs to measure corresponding autoantibodies using 87 breast cancer patients and 87 normal serum samples. A logistic regression model and leave-one-out validation were used to evaluate predictive accuracies with a single marker as well as with combined markers. Identities of those selected proteins were revealed through the sequence BLAST program.

**Results:**

We harvested 100 putative tumor-associated phage clones after biopan enrichment. Sequencing analysis revealed that six phage proteins were inframe and unique. Antibodies to these six phage-expressed proteins were measured by ELISAs, and the results showed that three of the phage clones had statistical significance in discriminating patients from normal individuals. BLAST results of the three proteins showed great matches to ASB-9, SERAC1, and RELT. Measurements of the three predictive phage proteins were combined in a logistic regression model that achieved 80% sensitivity and 100% specificity in prediction of sample status, whereas leave-one-out validation achieved 77.0% sensitivity and 82.8% specificity among 87 patient samples and 87 control samples. Receiver operating characteristic curve analysis and the leave-one-out method both showed that combined measurements of the three antibodies were more predictive of disease than any of the single antibodies studied, underscoring the importance of identifying multiple potential markers.

**Conclusion:**

Serum autoantibody profiling is a promising approach for early detection and diagnosis of breast cancer. Rather than one autoantibody, a panel of autoantibodies appears preferable to achieve superior accuracy. Further refinements will need to be made to further improve the accuracy. Once refined, the assay must be applied to a prospective patient population to demonstrate applicability.

## Introduction

In women, breast cancer is the most common malignancy and the second most common cause of cancer-related mortality [1]. Further reduction in the mortality will require successful strategies for early detection and screening of the disease. A sensitive assay to identify biomarkers that can accurately determine the onset of breast cancer – especially if the technique is of low risk to the patient, such as blood drawing – is ideal for early cancer detection.

Much of the effort in the past has centered on the discovery and characterization of single tumor-associated antigens as cancer markers. Two clinically used breast cancer antigens, CA 15-3 and CA 27.29, are elevated in less than 10% of early-disease patients and in about 75% of advanced-disease patients. Neither antigen is recommended for screening or diagnosis of onset breast cancer [2]. On the basis of the marked heterogeneity of most human cancers, it is doubtful that a single gene, chromosome aberration, or protein will provide sufficient accuracy for early detection.

In contrast to the detection of serum antigens, the detection of serum antibodies to tumor antigens may provide reliable serum markers for cancer diagnosis and prognosis [3-6]. Changes in the level of gene expression [3,7-9] and aberrant expression of tissue-restricted gene products [10,11] are factors in the development of a humoral immune response in cancer patients. There are several advantages of using serum antibodies as markers for tumor development. First, tumor-associated autoantibodies circulate in the blood much earlier than serum antigens. Autoantibodies to p53 have been reported in patients with early-stage ovarian or colorectal cancers [12,13], and a panel of serum antibodies can detect nonsmall-cell lung cancer 5 years prior to autoradiograph detection [14]. Second, antibodies may be more abundant than antigens, especially at low tumor burden. Thirty percent of patients with ductal carcinoma *in situ *in which the proto-oncogene HER-2/*neu *was overexpressed had serum antibodies specific to this protein [7,15]. In this respect, serologic analysis of recombinant cDNA expression libraries of tumors with autologous serum has identified some relevant tumor antigens: MAGE [16], SSX2 [17], and NY-ESO-1 [18].

We previously reported the use of combined autoantibodies as markers for early detection of nonsmall-cell lung cancer [14,19,20]. We achieved over 90% sensitivity and specificity in diagnosing stage I nonsmall-cell lung cancer using five antibody markers in serum samples. Can the previously described technique be applied to breast cancer? If so, can the technique provide sufficient sensitivity and specificity to be clinically useful?

In the present study, we used similar techniques on a breast cancer T7 cDNA phage library to identify tumor-associated proteins. We then measured autoantibody reactivities to identified phage-expressed proteins in breast cancer patient sera and normal sera by immunochemistry and ELISA to evaluate the sensitivity and specificity of single versus combined antibody measurements for predicting probability of disease. We further present the identities of corresponding proteins and their relevance to tumor biology.

## Methods

### Human subjects

After investigational review board approved and informed consent was obtained, 87 serum samples were obtained from individuals with histologically confirmed stage I to stage III breast cancer patients (11 stage I patients, 28 stage II patients and 48 stage III patients). Eighty-seven normal control serum samples were obtained from age-matched and sex-matched cohorts. All of the samples were collected from Hebei University Affiliated Hospital.

### Phage-display and biopanning process

A T7 phage breast cancer cDNA library (Novagen, Madison, WI, USA) was biopanned with sera from five pooled breast cancer patients and five pooled normal healthy donors, to screen potential autoantigens recognized by circulating antibodies in patient sera as previously described [19]. Briefly, the phage-displayed library was affinity-selected by incubation with protein G-agarose beads (Santa Cruz Biotechnology, Santa Cruz, CA, USA) coated with antibodies from pooled normal sera (250 μl pooled normal sera, diluted 1:20, at 4°C overnight) to remove nontumor-specific proteins. Unbound phages were separated from phages bound to antibodies in normal plasma by centrifugation. The supernatant was then biopanned against protein G-agarose beads coated with pooled patient plasma (4°C overnight) and separated from unbound phages by centrifugation. The bound/reactive phages were eluted with 1% sodium dodecyl sulfate and centrifugation. The phages were amplified in *Escherichia coli *BLT5615 (Novagen) in the presence of 1 mM isopropyl-β-D-thiogalactopyranoside and carbenicillin (50 μg/ml) until lysis. Amplified phage-containing lysates were collected and subjected to three additional sequential rounds of biopan enrichment. Bacterial cells were lysed by phage growth and yielded titers of 3 × 10^9 ^plaque-forming units/ml enriched phages.

### Immunodetection of antigen-expressing phages

To confirm the selection of four rounds of the biopanning procedure, limiting dilutions of phages from biopan 4 were used to infect bacteria and were grown on a LB-Agar plate covered with 6% agarose (LB-Agar/agarose). Phage plaques were lifted twice by placing two nitrocellulose disk membranes (Millipore, Billerica, MA, USA) onto plaque-formed LB-Agar/agarose plates (4°C, 2 hours. The lifted membranes were washed with 1 × Tris-buffered saline plus 0.1% Tween 20 and were blocked with 5% dry milk in 1 × Tris-buffered saline/Tween 20 for 1 hour at room temperature.

One membrane was probed with five pooled breast cancer patient sera, and the other membrane was probed with five pooled normal sera (1:7,500). Both patient sera and normal sera were the same as those used in the previous biopans. The membranes were followed by anti-human horseradish peroxidase-conjugated secondary antibody (1:10,000; Jackson ImmunoResearch, West Grove, PA, USA). The images were detected with electrogenerated chemiluminescence (TianGen Biotech, Beijing, China).

These two identical membranes allow a direct comparison of the immunoreactivity of individual phage clones with pooled patient and normal sera used in the biopan. Colonies corresponding to highly immunoreactive spots on the membrane were harvested from the original LB-Agar/agarose plate and amplified in *E. coli *as previously described.

### Sequencing and identification of phage-displayed tumor-associated proteins

Phage clones were isolated as above and the cDNA inserts were PCR-amplified using commercially available T7 phage vector primer (Novagen). The sequences are: T7 up, 5'-GGAGCTGTCGTATTCCAGTC-3'; and T7 down, 5'-AACCCCTCAAGACCCGTTTA-3'. Sequences of unique clones were checked for the open reading frame (ORF) status in the T7 expression vector. Only the correct ORF-encoded proteins were identified by comparison with known sequences in the GenBank database using the BLAST search program [21].

### Measurement of antibodies to phage-expressed proteins

ELISAs were developed for the identified inframe phage-expressed proteins to evaluate their immunogenic reactivity with different patient serum. Ninety-six-well microtiter ELISA plates (Jet Biofil, Guangzhou, China) were separately coated with the identified ORF tumor-associated proteins or empty phages as a negative control (2.5 × 10^10 ^phage/well in 1 × PBS/0.1% BSA at 4°C overnight, were blocked (PBS/1% BSA 37°C × 1 hour) and were washed (PBS/Tween 20). Serially diluted (1:20 to 1:10,240) serum samples from individual patients that were not used in the biopan were added to each well (37°C × 1 hour), and the plates were washed and then incubated with anti-human horseradish peroxidase secondary antibody (37°C × 1 hour). Assays were developed with tetramethyl benzidine/H_2_O_2 _substrate (Amresco, Solon, OH, USA) and stopped with 2 M H_2_SO_4_, and were then read on a spectrophotometer at 450λ. Each individual serum was run in triplicate.

In separate experiments, sera were assayed at a single dilution (1:320), and absorbance was used as a measure of antibody reactivity in each independent assay. Eighty-seven patient serum samples and 87 normal serum samples were assayed for antibodies to the phages expressing inframe proteins. The data were analyzed both individually for each marker and in possible combinations of markers.

### Statistical analysis

Using a panel of inframe phage-expressed proteins, logistic regression analysis was performed to predict the probability that a sample was from a breast cancer patient using SAS statistical software (SAS, Cary, NC, USA). Data for all 174 samples (87 patient samples and 87 normal samples) elicited from the above ELISA test were randomly chosen to build up classifiers that were able to distinguish patient samples from normal samples on the basis of an individual marker or a combination of markers.

Receiver operating characteristic curves were generated to compare the area under the curve and the predictive sensitivity and specificity with different markers using JMP software (**SAS **Cary, NC, USA). The classifiers were further examined by leave-one-out cross-validation.

## Results

### Selection of tumor-associated phage-expressed proteins

**Figure 1:**
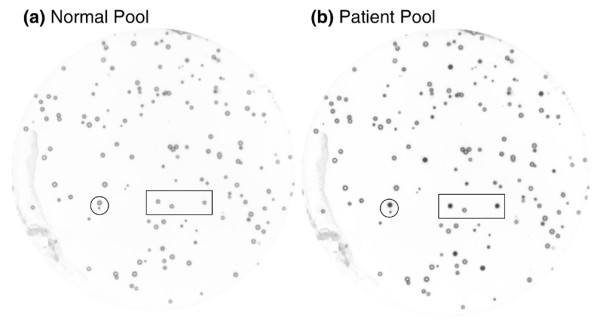
**Identification of disease-specific phage clones after the biopanning process**. Two nitrocellulose membrane disks were placed on and then lifted from the same phage grown plate of biopan 4. **(a) **One membrane was probed with pooled normal sera and **(b) **the other was probed with pooled patient sera. After electrogenerated chemiluminescence detection, numerous immunoreactive clones showed more intensified spots on the membrane incubated with patient sera than on the membrane incubated with normal sera. The circle and square indicate the same area on the two membranes.

Comparison between duplicate plaque-lift membranes of biopan 4, which were incubated with pooled breast cancer patient sera or normal sera used in the biopan, showed the ability of the biopan process to select immunogenic phage-expressed proteins. Immunoreactivity of multiple phage-expressed clones exhibited high-affinity binding with antibodies in patient sera. In contrast, these same clones had low-affinity binding in the identical membrane incubated with normal serum. The background seen on the membrane incubated with the normal sera was considered nonspecific reactivity with phage proteins (Figure 1).

One-hundred putative tumor-associated clones (darker spots) were selected for PCR and sequencing analysis. Among the 100 clones, six were found unique and expressing inframe proteins, and 16 phage clones were found redundant and 78 clones were incorrect ORF with the T7 phage vectors. DNA sequences of these six inframe proteins were then BLAST against the GenBank database and homologies were found to the following proteins: KLF17, COL6A1, GRWD1, ASB-9, SERAC and RELT. The complete names and known functions of the proteins are presented in Table 1.

**Table 1:** Proteins identified by biopanning from a breast cancer cDNA T7 phage library

Protein	Full name; functions	Score (bits), E^a ^value (alignment)
KLF17	Kruppel-like factor 17; new member of the Sp/KLF family of transcription factors in breast and prostate cancer	553, 1*e*-121 (100%)
COL6A1	Collagen, type VI, alpha 1; breast cancer and prostate cancer prognosis	626, 1*e*-143 (100%)
GRWD1	glutamate-rich WD repeat containing 1; overexpression in lung cancer, gastric cancer, and melanoma	545, 1*e*-152 (100%)
ASB-9	Ankyrin repeat and SOCS box protein 9; overexpression in breast cancer and prostate cancer	608, 1*e*-171 (100%)
SERAC1	Serine active site containing 1; unknown function	460, 3*e*-127 (100%)
RELT	Receptor expressed in lymphoid tissues; stimulating T-cell proliferation in the presence of CD3 signaling	422, 1*e*-115 (100%)

^a^The Expect value (E) is a parameter that describes the number of hits one can "expect" to see by chance when searching a database of a particular size.

### Antibody affinity for phage-expressed proteins

**Figure 2:**
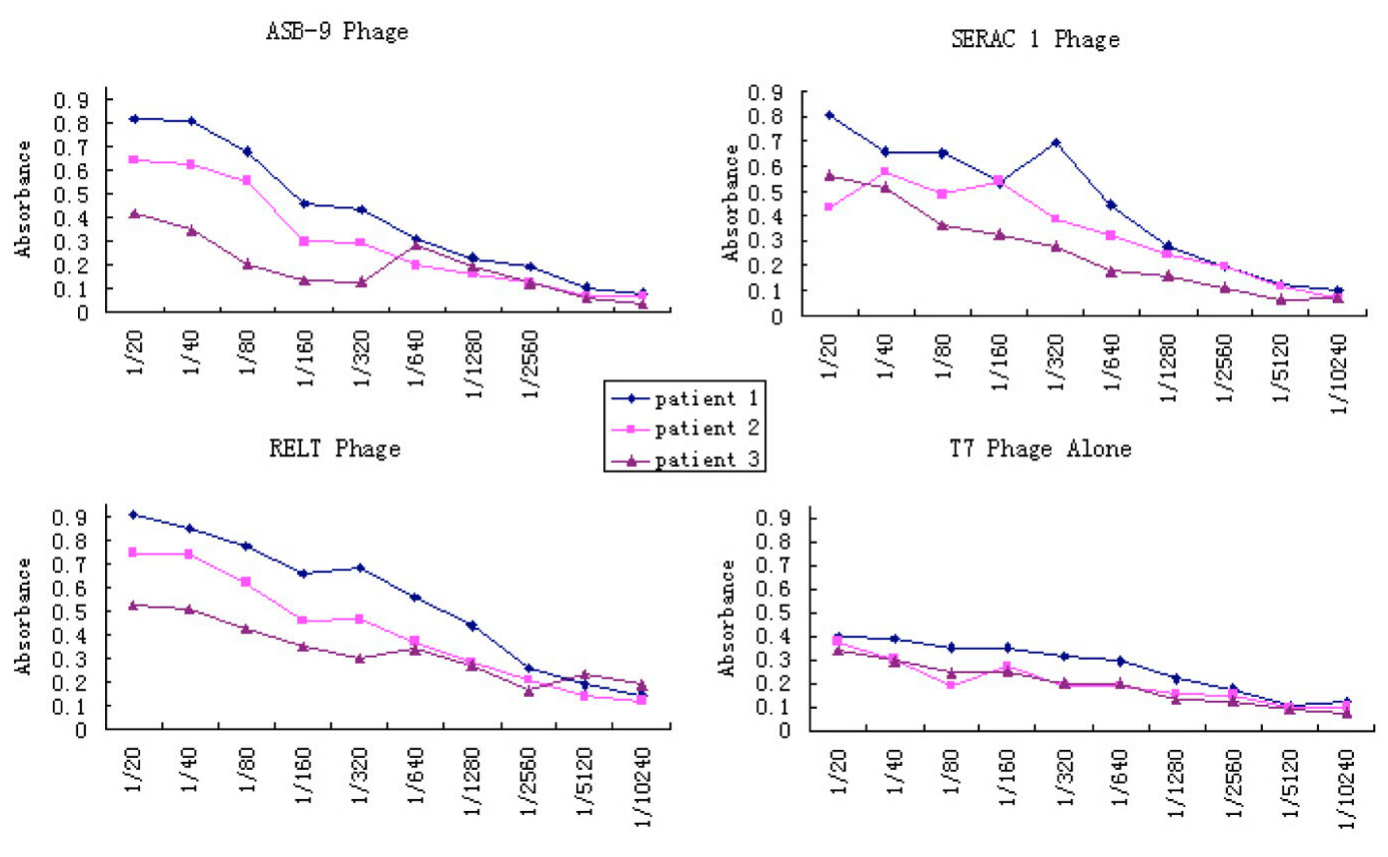
**ELISA of phage-expressed proteins with individual serum samples**. Antigen ELISAs were developed with ASB-9-expressing, SERAC1-expressing and RELT-expressing phages. The assays were performed with serially diluted (1:20 to 1:10,240) individual serum samples that were not used in the biopan, to confirm measurements were representative of an antigen-antibody affinity reaction. Representative curves from three patients are shown for each protein. Empty (no inserts) T7 phages were used to show the nonspecific reaction backgrounds.

To confirm antibody affinity in individual serum samples for specific proteins, serum was assayed in limiting dilution from 1:20 to 1:10,240 by ELISA constructed with phages ASB-9, SERAC1 and RELT, and T7 empty phages as control. Absorbance values for each of the three antibodies showed decreasing absorbance over serial dilutions in sera of three patients, indicating the antigen-antibody binding affinities. T7 empty phage controls exhibited background signals to patient sera (Figure 2).

### Measurement of antibody activities to open reading frame phage-expressed proteins

To assess the diagnostic potential of single antibody versus combined antibody measurements, the six inframe phage-expressed proteins KLF17, COL6A1, GRWD1, ASB-9, SERAC1, and RELT were developed in ELISAs to measure corresponding antibodies in individual patient sera and normal sera. Logistic regression was used to model the probability that a serum sample was from a breast cancer patient.

First, each of the six markers was included in the model individually. RELT was the most significant (*P *= 0.0001) with an area under the curve equal to 0.727, and the optimal predictive accuracy was achieved with 53% sensitivity and 100% specificity; whereas KLF17, COL6A1 and GRWD1 showed no statistical significance (*P *= 0.091, *P *= 0.074 and *P *= 0.066, respectively) (Table 2).

**Table 2:** Logistic regression analysis

Protein	Area under the curve	Specificity (%)	Sensitivity (%)	*P *value
KLF17^a^	0.474	100	27.3	0.0912
COL6A1^a^	0.482	100	32.7	0.0743
GRWD1^a^	0.4903	100	35.1	0.0656
ASB-9	0.593	100	41.2	0.0112
SERAC1	0.642	100	47.1	0.0009
RELT	0.727	100	52.9	0.0001
Three combined	0.861	100	80	0.0001

Area under the receiver operating characteristic curve indicates the diagnostic accuracy of biomarkers; the highest area = 1. ^a^These phage-expressed proteins showed no statistical significance in distinguishing patient samples from normal samples either individually or in combination.

**Figure 3:**
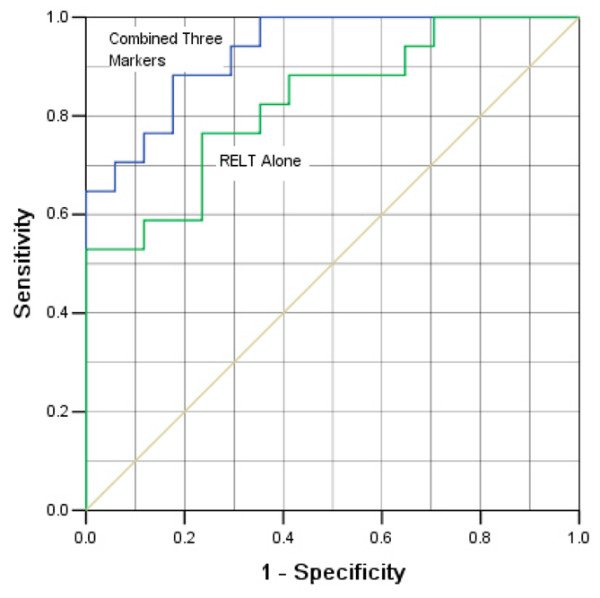
**Comparisons of the specificity and sensitivity of logistic regression models**. Data from quantitative ELISAs for three antibodies were evaluated for ability to predict disease. Lower curve: predictive accuracy using the logistic regression model with RELT data alone from 87 patients and from 87 normal persons as the explanatory variable. The area under the curve is 0.727 and the model is significant (*P *= 0.0001). Upper curve: predictive accuracy with the combination of ASB-9, SERAC1, and RELT as explanatory variables, where *P *= 0.0001 and the area under the curve is 0.861.

Next, different combinations among the six markers were tested in the model to determine the most optimal predictive values. The combination of ASB-9, SERAC1 and RELT was found to be the best grouping that produced an area under the curve equal to 0.861, and 80% sensitivity and 100% specificity (Figure 3).

To further validate the results, leave-one-out cross-validation was carried out with the same data used above, and both the individual and combined sensitivity and specificity were decreased (Table 3). The most predictive value of the three combined markers after the validations was 77.0% sensitivity and 82.8% specificity.

**Table 3:** Leave-one-out validation^a^

Protein	Specificity (%)	Sensitivity (%)	Diagnostic accuracy^b ^(%)
ASB-9	64.7	58.5	61.8
SERAC1	70.6	52.9	61.7
RELT	76.5	64.7	70.6
Three combined	82.8	77.0	79.9

^a^One sample was removed from the statistical model containing a total of 174 samples and a classifier was generated to predict the status (normal or patient) of the removed sample using the rest of the samples. This procedure was repeated for all samples. ^b^Diagnostic accuracy = (number of true positive + number of true negative)/total number of samples.

### Correlation of disease stages and diagnostic accuracies

To evaluate the ability of our assay in detection of early-stage breast cancer patients, we analyzed our data in association with the stages of the disease. After leave-one-out validation, the results showed 54.5% sensitivity on detection of stage I breast cancer patients, 75.0% sensitivity on stage II patients, and 83.3% sensitivity on stage III patients (Table 4). These results demonstrated that our combined biomarkers have greater sensitivity and specificity in predicting breast cancer patients than the traditional biomarkers.

**Table 4:** Diagnostic accuracies in control and different stage disease samples

	Matched control (*n *= 87)	Cancer (*n *= 87)		
		
		Stage I	Stage II	Stage III
Number correct/total	72/87	6/11	21/28	35/43
Accuracy (%)	82.8	54.5	75.0	83.3

## Discussion

Serum tumor markers have the potential of being incorporated into diagnostic and therapeutic practice in breast cancer [22-26]. Potential usages of the markers include early detection or screening, differentiation of benign from malignant disease, histological differentiation, and defining prognosis. These goals have generated considerable interests in identifying predictive tumor markers over the past three decades [27,28].

Besides CA 15-3 and CA 27.29, other breast tumor biomarkers have been identified. The most extensively investigated circulating biomarkers include MAGE family members, NY-ESO-1, HER-2/*neu*, MUC1, mutant p53, c-*myc*, BRCA1, and BRCA2. Depending on the stage and histology of disease, the percentage of breast cancer patients who have elevated serum protein levels of any one of the above markers is less than 36%, and combination of these markers ranges from 20% to 73% [22]. As such, the clinical application of these markers is limited when assayed independently, although using combinations of markers has somewhat enhanced diagnostic value [22]. Similarly, an antibody response to a single protein is not expected to be a universal marker [29-32].

In the present study, we interrogated a breast cancer cDNA T7 phage library with antibodies in patient sera to identify aberrantly expressed tumor proteins in breast cancer. Six correct ORF phage-expressed proteins were isolated and the corresponding antibody activities were measured by ELISA. The classifier using three combined phage markers has given good predictive accuracy using receiver operating characteristic curve analysis. Although this predictive accuracy was decreased by the leave-one-out validation from 174 samples, the value is still more sensitive and specific than any other biomarkers current used clinically.

We realized that the low percentage (6%) of ORF proteins of the T7 phage library we used in the present study had limited the number of inframe proteins that could be used for increasing the sensitivity and specificity. To conquer this problem, we have two potential solutions. First, we can choose a T7 phage library that was constructed using oligo-dT primer rather than random primers. Second, we can preselect the library by introducing second antibiotic genes at the C terminus of the expression vector [33].

Overall, our conclusion that multiple antibody measurements improve predictive accuracy is supported by the statistical analysis, although we still need to assay more serum samples against additional proteins to improve the statistical power and to validate this as a clinically reliable approach. Furthermore, the six sequences we identified in this study correspond to known proteins that cover a broad functional range, suggesting additional importance of this methodology for tumor biology and for expanding the number of potential targets for novel drug therapies and immunotherapies [34-43].

For diagnostic purposes, using an individual ELISA to measure more than a few antibodies would be cumbersome, and the measurement of all antibodies described here is impractical. Efficient alternatives to the ELISA could facilitate development of a highly predictive blood test for breast cancer. Fluorescent microarray technology, applied generally to gene discovery, and applied to high-throughput screening tumor-associated antigens, may be ideal for this purpose. Robotic microarray spotters that can group a comprehensive panel of phage-expressed proteins onto identical chips make it possible to reproducibly assay multiple antibodies in individual serum samples simultaneously. Double fluorescence used in protein microarray technology can decrease the false negative rate, and can facilitate normalization. Protein microarray technology is a powerful tool for screening tumor-associated antigens [20,38,39].

The tumor-associated antibodies we identified in breast sera have been described in other malignancies [40-43]. Given that several tumor-associated proteins we found in breast cancer are known to be expressed by other cancers, breast cancer specificity will have to be individually evaluated. Even assuming that no single putative marker is identified as being 100% specific for breast cancer, combined measurements of multiple markers may enhance the specificity. Testing a full panel of antibodies for breast cancer specificity will be an important part of assay validation and will eventually define clinical applicability.

Our data support the rationale for the development of an autoantibody panel for early detection and diagnosis of disease, and makes the identification of multiple proteins and their corresponding antibodies both logical and timely. In summary, the techniques used here to identify immunogenic proteins and their corresponding antibodies in peripheral blood might generate a panel or a profile of diverse markers that have significant diagnostic, therapeutic and scientific promise.

## Abbreviations

ELISA = enzyme-linked immunosorbent assay; BSA = bovine serum albumin; ORF = open reading frame; PBS = phosphate-buffered saline; PCR = polymerase chain reaction;

## Conclusion

In the present study we identified three breast tumor-associated proteins – ASB-9, SERAC 1 and RELT – from a breast cancer cDNA T7 phage library screening with antibodies in breast cancer sera. Through testing with 87 breast cancer patient serum samples and 87 control serum samples, the combined measurement of these three markers showed high sensitivity and high specificity for breast cancer detection. Our data indicate that antibody profiling is a promising approach that could achieve high diagnostic accuracy for breast cancer.

## Competing interests

The authors declare that they have no competing interests.

## Authors' contributions

LZ was involved in the experimental design, data interpretation and manuscript revision. KG carried out most of the experiments, data analysis and manuscript preparation. J-cZ made contributions to sample collection and manuscript revision. L-hZ was involved in data collection and manuscript preparation. W-kS made contributions to data analysis and statistical modeling. J-fW and X-gZ were involved in phage plaque assay and data acquisition. XG and WH were involved in sample collection and data analysis. YY and KHK were involved in data analysis and manuscript revision.
